# Case Report: Giant vulvar ulcer caused by Behcet's disease

**DOI:** 10.3389/fmed.2026.1902099

**Published:** 2026-07-30

**Authors:** Xin Li, Xianglong Zheng, Yuhuan Wang, Yaojie Chen, Moyan Chen, Hongcheng You, Wanchun Wang

**Affiliations:** 1 Clinical Medical College, Jiangxi University of Traditional Chinese Medicine, Nanchang, Jiangxi, China; 2 Affiliated Hospital of Jiangxi University of Traditional Chinese Medicine, Nanchang, Jiangxi, China

**Keywords:** Behcet's disease, case report, repair and reconstruction, skin flap transplantation, vulvar ulcer

## Abstract

**Rationale:**

Behcet's disease (BD) is a chronic, multisystem autoimmune vasculitis that can cause severe genital ulcers. Giant vulvar ulcers (diameter ≥4 cm) secondary to BD pose significant therapeutic challenges due to their refractory nature, risk of functional impairment, and impact on fertility and aesthetic outcomes.

**Patient concerns:**

A 19-year-old female presented with a left-sided vulvar ulcer that had persisted for over 4 months and worsened over the preceding 2 months.

**Diagnoses:**

The patient was diagnosed with a female vulvar ulcer secondary to Behcet's disease and perineal soft tissue infection based on the 2014 International Criteria for Behcet's Disease (ICBD).

**Interventions:**

The patient received intravenous glucocorticoid and antibiotic therapy, followed by pedicled skin flap transplantation, with adjuvant topical Shengji Yuyang ointment for wound care.

**Outcomes:**

Following systemic anti-inflammatory therapy, antibiotic treatment, and pedicled flap transplantation combined with topical Shengji Yuyang ointment, the ulcer healed completely within 6 weeks. At 5-month follow-up, no recurrence was observed. However, at month 6, after self-discontinuation of prednisone due to weight gain, a small recurrent ulcer developed, which resolved with topical corticosteroids and oral colchicine.

**Lessons:**

Vulvar ulcers secondary to BD may initially appear mild, but if diagnosis and treatment are delayed, they can rapidly progress, leading to extensive tissue destruction and impairment of genital aesthetics and reproductive function. Early identification and intervention are essential. Pedicled flap transplantation, when combined with adequate preoperative wound preparation, can both accelerate ulcer healing and preserve the aesthetic and functional integrity of the affected area.

## 1 Introduction

Behcet's disease (BD), also referred to as Behcet's syndrome (BS), is a chronic, multisystem autoimmune vasculitis. Its cardinal clinical features include oral ulcers, genital ulcers, ocular inflammation, and cutaneous lesions, with potential involvement of the nervous, vascular, and gastrointestinal systems (1). Oral ulcers usually present as an initial manifestation, while genital ulcers in females commonly affect the labia majora and minora and may extend to the vagina and cervix (2). The pathogenesis of BD remains incompletely understood; current evidence suggests a combination of genetic susceptibility, environmental triggers, and immunological factors (3). No specific laboratory test is available for BD, and diagnosis mainly relies on characteristic clinical manifestations. The co-occurrence of oral and genital ulcers constitutes the principal diagnostic criterion. According to the 2014 International Criteria for Behcet's Disease (ICBD) (Table 1), oral ulceration is not an absolute prerequisite for diagnosis; a total score ≥ 4 establishes the diagnosis of BD (4). Clinical management focuses on controlling inflammation, alleviating symptoms, and preventing irreversible organ damage (5). Corticosteroids, colchicine, and thalidomide are commonly employed for localized ulcer management, while patients with refractory disease may be candidates for interferon-α or canakinumab therapy (6, 7). Colchicine's mechanism mainly involves blocking leukocyte aggregation to the site of inflammation, reducing the release of oxygen free radicals and lysosomes, and alleviating local damage at the source. It is the first-line clinical treatment for preventing Behçet's disease recurrence.

**Table 1:** The 2014 International Criteria for Behcet's Disease (ICBD) scoring system.

Symptom and/or Sign	Score (points)
Oral ulceration	2
Ocular involvement	2
Genital ulceration	2
Skin lesions	1
Neurological involvement	1
Vascular involvement	1
Positive pathergy test	1

A total score ≥ 4 points is sufficient for the diagnosis of Behcet's disease.

We report a case of giant vulvar ulcer secondary to delayed diagnosis and treatment of BD. The patient was a 19-year-old woman whose vulvar ulcer progressively worsened, causing substantial damage to the aesthetics and functionality of the external genitalia. She recovered after systemic anti-inflammatory and antibiotic therapy combined with skin flap transplantation. This case describes the clinical course of a giant vulvar ulcer secondary to BD and the multidisciplinary management approach adopted, highlighting diagnostic and therapeutic considerations that may inform similar cases.

## 2 Case presentation

A 19-year-old Han Chinese female presented to our department on March 4, 2025, with a skin ulcer on the left vulva that had persisted for over 4 months and worsened over the preceding 2 months. Approximately 4 months prior to presentation, the patient had developed a papulopustular lesion near the vaginal orifice on the left vulva without a clear precipitating factor, accompanied by burning and pain. She sought medical attention at the university health center and received oral levofloxacin tablets and a topical ointment (details unknown) for anti-infection therapy; however, no significant improvement was observed. 2 months before presentation, the lesion ruptured, producing thick yellow purulent discharge mixed with blood, and the pain intensified. She presented to a local hospital where she underwent irrigation, dressing changes, and subsequent chronic ulcer repair surgery. Postoperative recovery was unsatisfactory, and the ulcer area enlarged further after suture removal. She was therefore referred to our hospital for further management. Throughout the disease course, she had remained conscious with fair general condition, normal physical strength, adequate appetite and sleep quality, and normal urination and defecation (an indwelling urinary catheter was in place with patent drainage). No significant changes in body weight were noted.

### 2.1 Past medical history

The patient was previously healthy, with no history of autoimmune diseases, no sexual activity, and recurrent oral ulcers. There was no family history of similar conditions.

### 2.2 Physical examination

**Figure 1:**
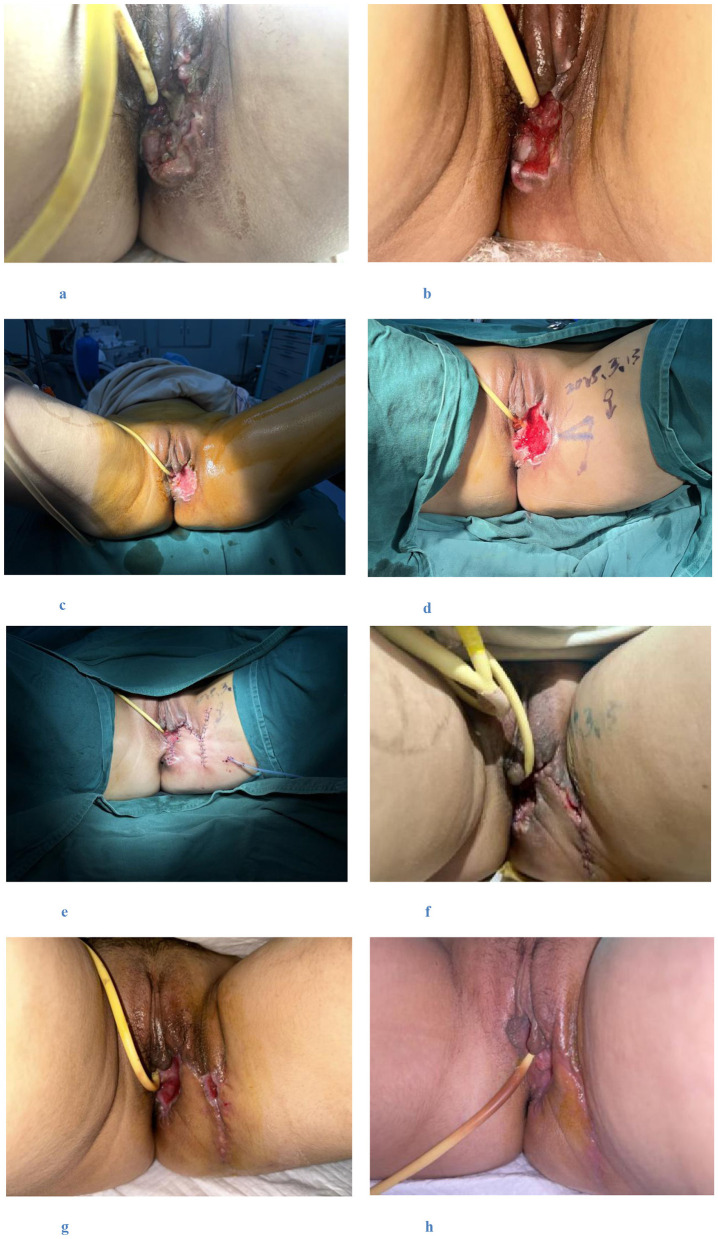
Complete healing process of the vulvar ulcer. **(a)** Wound condition on the day of admission (March 4). **(b)** Wound condition after 7 days of dressing changes (March 11). **(c–e)** Pre- and intraoperative views (March 13). **(f)** Partial suture disruption during defecation (March 21). **(g)** Wound condition on April 1. **(h)** Wound condition on April 12.

Systemic examination was unremarkable except for left inguinal lymphadenopathy. Dermatological examination revealed partial ulceration and loss of the left labia majora and minora, with an ulcer measuring approximately 3 × 4 × 2 cm (Figure 1a). The granulation tissue on the ulcer surface was rough with large, irregular granular protrusions, dark red in color, with visible necrotic tissue and thin white purulent exudate. Flocculent discharge and severe tenderness were present. The ulcer margins were dark purple, and the perilesional skin was erythematous and edematous.

### 2.3 Laboratory and auxiliary examinations

Complete blood count revealed a white blood cell count of 11.04 × 10^9^/L and a neutrophil count of 8.79 × 10^9^/L. C-reactive protein was elevated at 45.6 mg/L. Liver and renal function, electrolyte panel, cardiac enzyme profile, antinuclear antibody spectrum, rheumatoid factor triad, blood glucose, lipid profile, urinalysis, stool routine, HIV/syphilis serology, and hepatitis panel were all within normal limits. Bacterial culture and sensitivity testing indicated Escherichia coli infection, resistant to levofloxacin but sensitive to piperacillin/tazobactam. Non-contrast perianal magnetic resonance imaging revealed partial soft tissue loss in the perineum, with patchy soft tissue edema involving the perineum and pelvic floor, extending to the lower segment of the left anal canal and the lower vagina.

### 2.4 Histopathological examination of the vulvar lesion

**Figure 2:**
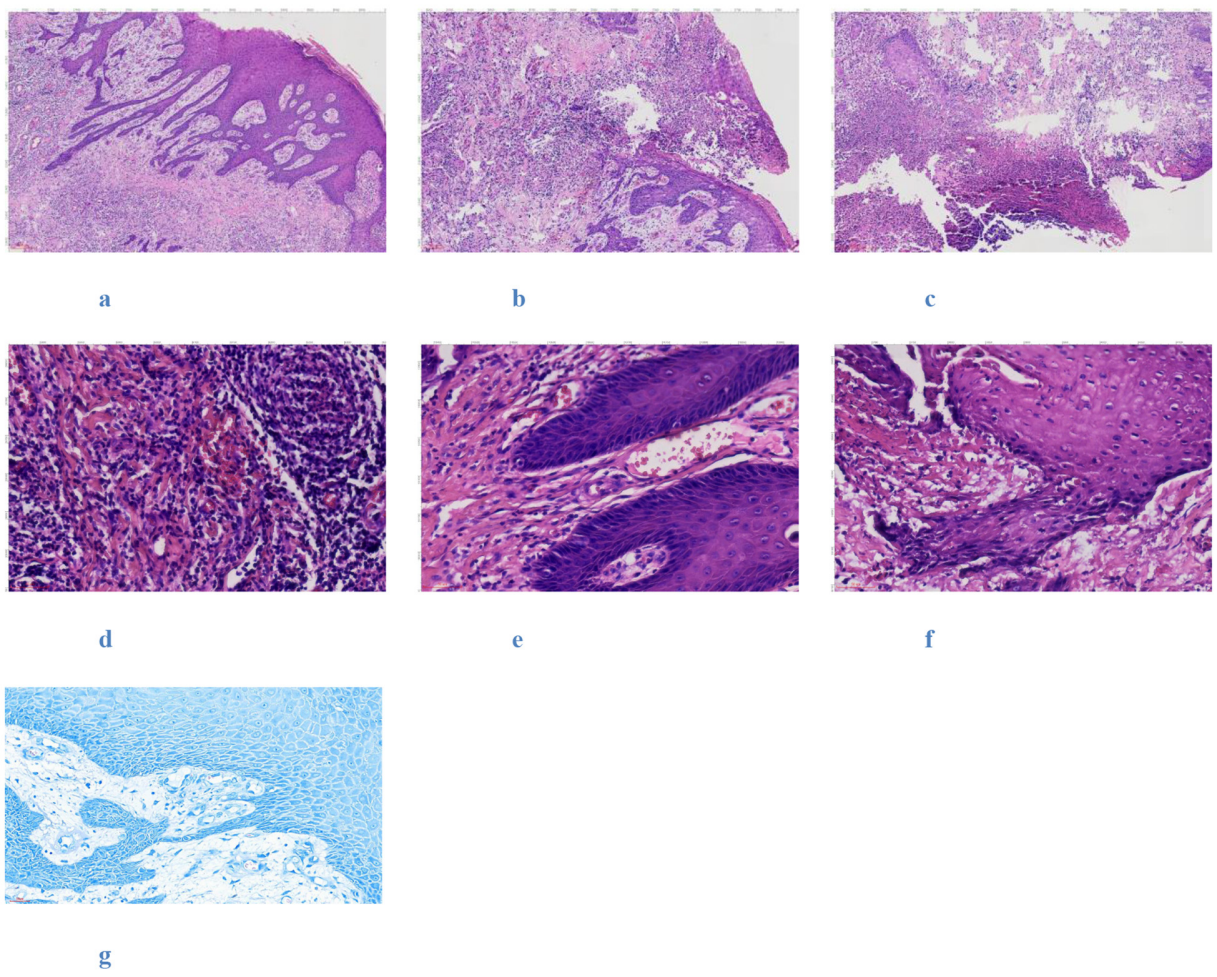
Histopathological images of the vulvar ulcer lesion. **(a–c)** H&E staining, objective magnification × 10 (scale bar = 200 μm). **(d–f)** H&E staining, objective magnification × 40 (scale bar = 50 μm). **(g)** Acid-fast staining, objective magnification × 20 (scale bar = 30 μm).

Showed findings consistent with ulceration accompanied by squamous epithelial pseudoepitheliomatous hyperplasia. Proliferating epithelial cells maintained good polarity, with no obvious atypia, pathological mitotic features, or interstitial infiltration, clearly excluding squamous cell carcinoma and conforming to reactive benign proliferative changes (Figures 2a–f). Acid-fast staining was negative, and no mycobacteria were identified within the examined fields (Figure 2g).

### 2.5 Diagnosis

(1) Female vulvar ulcer; (2) Behcet's disease; (3) Perineal soft tissue infection.

### 2.6 Treatment

After admission on March 4, 2025, we initiated intravenous piperacillin sodium-tazobactam sodium 4.5 g twice daily and intravenous dexamethasone sodium phosphate 5 mg once daily. Following wound disinfection, the topical traditional Chinese medicine preparation Shengji Yuyang ointment was applied to the affected area (Figure 1a). On March 11, after 7 days of dressing changes, the necrotic tissue on the ulcer surface had completely liquefied and sloughed. The granulation tissue appeared as fine granular projections, bright red in color, with no exudate and significantly reduced pain. Shengji Yuyang ointment was continued (Figure 1b). Dexamethasone sodium phosphate was switched to oral prednisone acetate 10 mg three times daily, with the dose gradually tapered. On March 13, we performed pedicled flap transplantation (Figures 1c–e). On March 19 (postoperative day 7), the surgical incision showed well-aligned edges with no exudate. No erythema or edema was observed at the wound margins, and no significant tenderness was elicited. Routine dressing changes were continued. On March 21, the patient inadvertently disrupted several sutures near the vaginal orifice and the medial thigh during defecation, resulting in partial wound dehiscence. Shengji Yuyang ointment was reapplied topically (Figure 1f). April 1 wound situation (Figure 1g). By April 12, the wound had completely healed (Figure 1h). The patient was discharged with instructions to continue oral prednisone acetate 2.5 mg once daily. During the first 5 months of follow-up, no recurrence was observed. In the 6th month, the patient discontinued the medication due to excessive weight gain attributed to long-term corticosteroid use, and a pea-sized (approximately 1 cm) ulcer recurred on the vulva. She was instructed to take oral colchicine 0.5 mg once daily and apply triamcinolone acetonide cream topically to the ulcer. The lesion healed, and no further recurrence has been observed (Table 2).

**Table 2:** Timeline of wound condition and treatment.

Time	Wound condition	Treatment
March 4, 2025	Partial ulceration and loss of the left labia majora and minora, with an ulcer measuring approximately 3 cm × 4 cm × 2 cm. The granulation tissue on the ulcer surface was rough with large, irregular granular protrusions, dark red in color, with visible necrotic tissue and thin white purulent exudate. Flocculent discharge and severe tenderness were present. The ulcer margins were dark purple, and the perilesional skin was erythematous and edematous (Figure 1a).	Intravenous piperacillin sodium-tazobactam sodium 4.5 g twice daily, intravenous dexamethasone sodium phosphate 5 mg once daily, topical Shengji Yuyang ointment
March 11, 2025	The necrotic tissue on the ulcer surface had completely liquefied and sloughed. The granulation tissue appeared as fine granular projections, bright red in color, with no exudate and significantly reduced pain (Figure 1b).	Dexamethasone sodium phosphate was switched to oral prednisone acetate 10 mg three times daily, topical Shengji Yuyang ointment
March 13, 2025	The granulation tissue on the ulcer surface appeared as fine granular projections, bright red in color, with no exudate and no significant pain (Figure 1c).	Pedicled flap transplantation was performed
March 19, 2025	The surgical incision showed well-aligned edges with no exudate. No erythema or edema was observed at the wound margins, and no significant tenderness was elicited.	Oral prednisone acetate 5 mg once daily, topical compound polymyxin B ointment
March 21, 2025	Partial suture disruption near the vaginal orifice and the medial thigh during defecation, resulting in partial wound dehiscence (Figure 1f).	Topical Shengji Yuyang ointment
April 12, 2025	The wound had completely healed (Figure 1h).	Oral prednisone acetate 2.5 mg once daily
October 5, 2025	One month after self-discontinuation of medication, a pea-sized (approximately 1 cm) ulcer recurred on the vulva.	Oral colchicine 0.5 mg once daily, topical triamcinolone acetonide cream

Figure references correspond to the images in the original case report.

## 3 Discussion

Female vulvar ulcer is a relatively common surgical condition with diverse etiologies, including infections, autoimmune diseases, trauma, and drug reactions. Clinically, vulvar ulcers are most commonly associated with syphilis, genital herpes, and malignant neoplasms. They typically present as disruption and denudation of the vulvar skin and mucosa, which may extend to the deep dermis or beyond, accompanied by severe pain and exudate, significantly impairing daily life (8). Vulvar ulcers with a maximum diameter ≥ 4 cm are classified as giant ulcers; the present case had a lesion measuring 4 cm at its longest dimension, thereby meeting the classification criteria for giant vulvar ulcers (9). Given the anatomical sensitivity and the extensive ulcerated surface, giant vulvar ulcers frequently compromise aesthetic outcomes and reproductive function (10). During clinical evaluation, the morphological appearance, surface area, and character of exudate provide preliminary clues for differentiating sexually transmitted from non-sexually transmitted ulcers and should be further corroborated by laboratory investigations (11). Notably, BD has no specific laboratory diagnostic marker; its diagnosis and differentiation rely primarily on clinical heterogeneity. Early identification and diagnosis are critical for improving the clinical course of the disease.

Acute vulvar ulcer (Lipschütz ulcer, LU), also known as acute genital ulcer, was first reported in 1912. It is a non-sexually transmitted disease whose pathogenesis is still unclear, but recent studies suggest that the occurrence of LU is associated with the COVID-19 vaccine (12). Clinically, nearly 90% of cases occur in young female under 20 years old (especially sexually inactive individuals) in the vulva or superficial vagina. The onset is abrupt, with single or multiple locally painful ulcers present. Gray necrotic or purulent centers may be seen on the ulcer surface, with abundant exudation of secretions, often symmetrically distributed, accompanied by inguinal lymph node enlargement. The vast majority of cases are associated with influenza-like or infectious mononucleosis syndrome, usually healing within 3 weeks (13). There are currently no laboratory markers for definitive diagnosis of LU. The main differentiation from genital ulcers caused by BD is that LU only presents with vulvar ulcer symptoms, without accompanying symptoms such as oral ulcers or conjunctivitis, and is self-healing, with no recurrence after stopping the medication (14).

The present patient had a prolonged disease course and a history of recurrent oral ulcers. Based on the clinical manifestations, physical findings, and relevant laboratory investigations, we established the diagnosis of a giant vulvar ulcer secondary to BD. According to the International Behçet's Disease Standards (ICBD) scoring system, the patient had recurrent oral ulcers (2 points) and genital ulcers (2 points), with a total score of 4 points. According to ICBD criteria (total score ≥4), this patient meets the diagnostic criteria for Behçet's disease. The ulcer had progressed to its extensive dimensions as a consequence of delayed diagnosis and inadequate prior treatment. For treatment, we selected corticosteroids for their rapid and potent anti-inflammatory effects (15). Based on the results of bacterial culture and susceptibility testing, we chose piperacillin sodium-tazobactam sodium as the targeted antibiotic, effectively eradicating the causative pathogen (Escherichia coli). Topical application of Shengji Yuyang ointment promoted debridement, resolution of necrotic tissue, activation of local blood circulation, and stimulation of tissue regeneration. During the early phase of ulceration, the ointment gradually cleared necrotic tissue through liquefaction and sloughing, fully exposing the fresh granulation tissue at the wound base and promoting its proliferation. During the later phase, as necrotic tissue separated and new tissue formed, Shengji Yuyang ointment upregulated the levels of basic fibroblast growth factor (bFGF) and epidermal growth factor (EGF) within the wound, thereby accelerating wound healing (16). Shengji Yuyang ointment may represent a potential adjunctive option for local wound bed preparation in the management of chronic ulcers, but its specific contribution remains unquantified and the evidence from this single case is insufficient to support a general treatment recommendation. Ulcers caused by BS typically resolve with conventional immunosuppressive and local anti-infection therapy. However, in this case, the ulcer was extensive, penetrating into the muscular and fascial layers. Furthermore, the patient was only 19 years of age and had not yet married and conceived. Natural healing would likely entail a prolonged course with extensive scar formation and poor tissue pliability, potentially compromising aesthetic outcomes and reproductive function (1). After comprehensive consideration, we decided to perform pedicled flap transplantation once the necrotic tissue had completely sloughed, the granulation tissue appeared bright red, there was no significant exudate, and the wound base had become substantially smoother. This approach aimed to accelerate wound healing, restore local function, and improve aesthetic outcomes (17). BD relapses after stopping medication because peripheral Th1/Th17 memory lymphocytes remain latent for a long time in lymph nodes, mucosal lamina propria, and vascular adventitia. Circulating inflammatory factors can completely turn negative, but memory T cells are not cleared by hormones, thalidomide, or other drugs; they are merely suppressed and dormant (3, 5). BD's long-term maintenance strategy should be to use the lowest dose of medication to suppress immunity for extended periods, eliminate delayed relapse, and prevent severe symptoms such as thrombosis, intestinal disease, and optic nerve atrophy in the long term. If intolerance or drug-related adverse reactions occur during medication, other immunomodulatory drugs can be sequentially switched for maintenance therapy.

A limitation of this case was inadequate postoperative patient education, which led to inadvertent suture disruption during defecation, slowing the healing process and prolonging treatment duration. Although the patient had received general wound-care instructions at discharge, explicit guidance on safe defecation positioning and perineal wound protection during bowel movements was insufficiently emphasized; this gap has since been addressed in our perioperative counseling protocol. We propose the following strategies to prevent similar complications in future cases: (1) application of Steri-strips or wound closure tapes to reduce wound tension and protect the incision (18); (2) bedpan defecation to eliminate wound tension from squatting; (3) enteral nutritional formulations to reduce bowel movement frequency while meeting nutritional requirements (19, 20). Each strategy has its merits and limitations, and the optimal approach should be individualized based on patient circumstances and further refined through clinical experience. Notably, the patient experienced a recurrence at 6 months after self-discontinuation of corticosteroids, underscoring the chronic and relapsing nature of BD and the importance of gradual tapering under medical supervision rather than abrupt cessation. The recurrence responded well to colchicine and topical corticosteroids, consistent with the known efficacy of these agents for mucocutaneous BD lesions.

In conclusion, although giant vulvar ulcers caused by BD are uncommon, they exhibit characteristic clinical features. Timely diagnosis and appropriate treatment generally yield a favorable prognosis. However, if untreated or inadequately managed, these ulcers are prone to recurrence and progression, potentially causing irreversible damage to the external genitalia with consequent impairment of aesthetic and reproductive function. Pedicled flap transplantation not only promotes ulcer healing but also substantially improves both the aesthetic appearance and functional outcomes of the affected area.

## 4 Patient perspective

The patient expressed profound distress regarding the impact of the ulcer on her body image, daily activities, and future reproductive health. She reported that the persistent pain and disfigurement caused significant psychological burden and social withdrawal during the 4-month period before effective treatment. Following flap transplantation and complete healing, she reported marked improvement in quality of life and self-confidence. She emphasized that clear communication about postoperative precautions, particularly regarding defecation positioning, would have helped her avoid the setback of suture disruption. Overall, she expressed satisfaction with the final aesthetic and functional outcome.

## Data Availability

The original contributions presented in the study are included in the article/Supplementary material, further inquiries can be directed to the corresponding author.
