# Supplementary material for: Case Report: Giant vulvar ulcer caused by Behcet's disease
Source: Front Med (Lausanne). 2026 Jul 30;13:1902099. doi: 10.3389/fmed.2026.1902099 (PMC13467751; doi:10.3389/fmed.2026.1902099)
Supplement: Supplementary file 1 [file Supplementary_file_1.pdf]

Appendix:Differential Diagnosis of Vulvar Ulcer

★Behçet's disease is the confirmed diagnosis in the present case.The table is structured to facilitate systematic differential diagnosis of vulvar ulcers,with key distinguishing features highlighted for each condition.

Disease	Pathogen/Etiology	Ulcer Characteristics	Laboratory Diagnosis	Histopathology
**A.Sexually Transmitted Infections(STIs)**				
Primary Syphilis (Chancre)	Treponema pallidum	Solitary,well-demarcated<br>Indurated(hard)base<br>Painless,clean surface<br>Regional lymphadenopathy	RPR/VDRL/TPPA:reactive<br>Dark-field:motile spirochetes	Dense plasma cell infiltrate<br>Endarteritis obliterans<br>Warthin-Starry:spirochetes
Chancroid	Haemophilus ducreyi	Multiple,painful,soft-based<br>Irregular undermined edges<br>Purulent exudate<br>Inguinal bubo(~ 50%)	Culture:special media required<br>PCR:H.ducreyi DNA<br>Gram:"school of fish"pattern	Three-zone pattern:<br>1)Superficial necrosis<br>2)Granulation tissue<br>3)Deep plasma cells
Donovanosis	Klebsiella granulomatis	Painless,beefy-red<br>Rolled margins,granulomatous<br>Friable,bleeds easily<br>No lymphadenopathy	Tissue smear:Donovan bodies<br>PCR:K.granulomatis	Dense histiocytic infiltrate<br>Donovan bodies in macrophages
Necrotic HSV	HSV-1/HSV-2	Clustered vesicles→coalescing erosions/ulcers<br>Erythematous base<br>Immunocompromised:extensive	HSV PCR:gold standard<br>Tzanck:multinucleated giant cells<br>Serology:type-specific IgG	Ballooning degeneration<br>Cowdry type A inclusions<br>Multinucleated giant cells
LGV	Chlamydia trachomatis (L1–L3)	Small,transient,painless papule<br>Painful inguinal bubo<br>"Groove sign"	NAAT/PCR:C.trachomatis<br>Serology:CF≥1:64	Non-specific inflammation<br>Stellate abscesses in nodes
**B.Non-STI Infectious Causes**				
Amoebic Ulcer	Entamoeba histolytica	Irregular,painful<br>Undermined ragged edges<br>Necrotic base,bloody exudate<br>±Diarrhea/dysentery	Stool microscopy:trophozoites/cysts<br>Tissue smear:trophozoites with ingested RBCs(pathognomonic)	Trophozoites in tissue(PAS+)<br>±ingested erythrocytes
Cutaneous TB	Mycobacterium tuberculosis	Irregular,violaceous undermined edges<br>Granular base,scanty discharge<br>Chronic,slowly progressive	AFB smear/culture<br>PCR:Xpert MTB/RIF<br>TST/IGRA:positive	Caseating granulomas<br>Langhans giant cells<br>ZN/Fite:AFB positive
Deep Fungal Infection	Candida,Cryptococcus, Histoplasma,etc.	Variable;chronic indolent ulcer<br>Irregular borders,necrotic base<br>Immunocompromised host risk	KOH preparation<br>Fungal culture:Sabouraud<br>Serumβ-D-glucan	Yeast/pseudohyphae/hyphae<br>PAS&GMS:positive
Bacterial Infection (Non-STI)	S.aureus,S.pyogenes, E.coli,anaerobes	Painful,purulent exudate<br>Erythema,edema,warmth<br>Often secondary to trauma	Bacterial culture&sensitivity<br>Gram stain of exudate/tissue	Non-specific acute inflammation<br>Neutrophilic infiltrate<br>Gram:identifies organisms
Lipschütz Ulcer	Reactive/post-infectious (EBV,CMV,influenza)	Acute onset,painful,deep<br>Well-demarcated,symmetric	EBV/CMV serology<br>Exclusion of STIs	Non-specific necrosis<br>Negative special stains

Disease	Pathogen/Etiology	Ulcer Characteristics	Laboratory Diagnosis	Histopathology
		Adolescent/young women<br>Self-limiting(2–6 wk)	Clinical diagnosis	
**C.Non-Infectious Inflammatory&Other Causes**				
Behçet's Disease★<br>(Confirmed in this case)	Systemic vasculitis<br>(Unknown etiology)	Recurrent oral+genital ulcers<br>Well-demarcated,aphthous-like<br>±Ocular,skin,vascular,<br>neurologic involvement<br>ICBD score≥4=diagnosis	Clinical diagnosis:ICBD criteria<br>Pathergy test:±positive<br>HLA-B51:associated<br>No specific laboratory marker	Leukocytoclastic vasculitis<br>Perivascular lymphocytic infiltrate<br>Non-specific:biopsy to exclude infection/malignancy
Pyoderma Gangrenosum(PG)	Autoimmune/ neutrophilic dermatosis	Rapidly progressive,very painful<br>Violaceous undermined borders<br>Purulent/necrotic base<br>Pathergy phenomenon<br>Associated:IBD,RA,hematologic dz	Diagnosis of exclusion<br>Biopsy to exclude infection<br>Colonoscopy:rule out IBD<br>Response to steroids supports dx	Neutrophilic dermal infiltrate<br>Abscess formation<br>Necrosis±vasculitis<br>CRITICAL:negative special stains
Crohn's Disease<br>(Metastatic)	Inflammatory/autoimmune	Linear"knife-cut"ulcerations<br>Cobblestoning appearance<br>Perineal/vulvar involvement<br>±GI symptoms	Colonoscopy+biopsy:<br>Non-caseating granulomas<br>Fecal calprotectin:elevated	Non-caseating granulomas<br>Transmural inflammation<br>Lymphoid aggregates
Malignant Ulcer (SCC)	Neoplastic (HPV,lichen sclerosus)	Chronic,non-healing<br>Irregular,raised everted edges<br>Indurated,firm base<br>Risk factors:HPV,LS,smoking	Biopsy:gold standard<br>HPV typing:high-risk types<br>Imaging:MRI/CT for staging	Atypical squamous cells<br>Invasive nests<br>Keratin pearls<br>Nuclear pleomorphism

Abbreviations:AFB=acid-fast bacilli;CF=complement fixation;CMV=cytomegalovirus;CRP=C-reactive protein;CT=computed tomography;EBV=Epstein-Barr virus;GMS=Gomori methenamine silver;HLA=human leukocyte antigen;HPV=human papillomavirus;HSV=herpes simplex virus;IBD=inflammatory bowel disease;ICBD=International Criteria for Behçet's Disease;IGRA=interferon-gamma release assay;IHC=immunohistochemistry;LGV=lymphogranuloma venereum;LS=lichen sclerosus;MRI=magnetic resonance imaging;NAAT=nucleic acid amplification test;PAS=periodic acid-Schiff;PCR=polymerase chain reaction;PG=pyoderma gangrenosum;RPR=rapid plasma reagin;SCC=squamous cell carcinoma;TPPA=Treponema pallidum particle agglutination;TST=tuberculin skin test;VDRL=Venereal Disease Research Laboratory;ZN=Ziehl-Neelsen.
